# Exploring Professionalism Dilemma and Moral Distress through Medical Students’ Eyes: A Mixed-Method Study

**DOI:** 10.3390/ijerph191710487

**Published:** 2022-08-23

**Authors:** Cordelia Cho, Wendy Y. K. Ko, Olivia M. Y. Ngan, Wai Tat Wong

**Affiliations:** 1 Faculty of Medicine, The Chinese University of Hong Kong, Hong Kong, China; 2 CUHK Centre for Bioethics, Faculty of Medicine, The Chinese University of Hong Kong, Hong Kong, China; 3 Department of Anaesthesia and Intensive Care, Faculty of Medicine, The Chinese University of Hong Kong, Hong Kong, China

**Keywords:** moral distress, professionalism, bioethics, medical student, medical education

## Abstract

This study aims to understand professionalism dilemmas medical students have experienced during clinical clerkships and the resulting moral distress using an explanatory mixed-method sequential design—an anonymous survey followed by in-depth interviews. A total of 153 students completed and returned the survey, with a response rate of 21.7% (153/706). The top three most frequently occurring dilemmas were the healthcare team answering patients’ questions inadequately (27.5%), providing fragmented care to patients (17.6%), and withholding information from a patient who requested it (13.7%). Students felt moderately to severely distressed when they observed a ward mate make sexually inappropriate remarks (81.7%), were pressured by a senior doctor to perform a procedure they did not feel qualified to do (77.1%), and observed a ward mate inappropriately touching a patient, family member, other staff, or student (71.9%). The thematic analysis based on nine in-depth interviews revealed the details of clinicians’ unprofessional behaviors towards patients, including verbal abuse, unconsented physical examinations, bias in clinical decisions, students’ inaction towards the dilemmas, and students’ perceived need for more guidance in applying bioethics and professionalism knowledge. Study findings provide medical educators insights into designing a professional development teaching that equips students with coping skills to deal with professionalism dilemmas.

## 1. Introduction

Professionalism dilemmas are pervasive, and the exposure of medical students to unethical behaviors persists and remains unsolved [1,2]. Common day-to-day challenges include performing examinations on sedated or anesthetized patients without consent, performing procedures with insufficient training, breaches of patient safety and dignity, administering drugs amidst patient refusal, discussing confidential information in inappropriate settings, and covering up mistakes [3,4,5]. In this article, we refer to professionalism dilemmas as the ethically problematic situations in healthcare settings that one finds improper, wrong, or unethical [6,7]. The inability to resolve difficulties or being forced to act against one’s conscience and values at the workplace is an antecedent of poor patient quality care and heightened levels of distress [8]. A theoretical framework identifying the emergence and ramifications of moral distress supports the interrelationship between morally challenging situation and moral distress [9].

Moral distress refers to the emotional discomfort that arises when one identifies a morally wrong situation but fails to take action due to institutional or hierarchical constraints [10]. It can be broadly categorized in a spectrum of three stages [11]: mild distress refers to a sole moment in which a person feels uneasy or upset; moderate distress refers to a feeling that continues for weeks or even months after an event; and severe distress refers to a circumstance where distress persist many months or even years. Moral distress penetrates at different levels across different disciplines. Situations that produce moral distress include aggressive treatment of dying patients, participating, or witnessing substandard care, breaches of patient safety and dignity, withholding information from patients, inadequate support, and inconsistencies between some aspects of medical education and clinical practice [4,12,13,14,15]. Research suggested the distress resulting from professionalism dilemmas induced long-lasting emotional episodes compromising mental well-being. Medical students felt burnout [14], emotional breakdown [15,16], and guilty [5,17] in the face of professionalism dilemmas. Some students have even dropped out of medical school or chosen a non-clinical specialty as a result [18]. At worst, one is likely to suffer from the “moral residue” ,which aggravates a cumulative effect in a future career when one fails to perform ethical actions in dilemmas [18,19,20].

Professionalism dilemmas are not exclusive to the Northern American or European counterparts and are reported in the Asia-Pacific region, including Bangladesh [21], Malaysia [22], Pakistan [23], and Singapore [24]. The receptivity to dilemmas is context-specific to social-cultural values, workplace environment, patient-physician relationship, hierarchy, and the healthcare system. This study is grounded on Wilkinson’s theoretical framework asserting that moral distress arises when one is faced with dilemmas in conflict with their beliefs, expectations, and values. To the best of our knowledge, there is limited information exploring professionalism dilemmas during the clerkship and to what extent medical and culture influence medical students’ responses. This study aims to understand professionalism dilemmas medical students have experienced during clinical clerkship. In line with the goal, the specific objectives are (1) to quantitatively assess the prevalence of witnessing or participating in professionalism dilemmas and examine the level of moral distress resulting from these experiences, and (2) to qualitatively explore professionalism dilemmas and related emotions observed during clinical clerkship.

## 2. Materials and Methods

### 2.1. Study Context

The present study was conducted at the Chinese University of Hong Kong, one of the two local medical schools offering a six-year undergraduate medical degree program [18]. The first three years are a pre-clinical clerkship emphasizing interdisciplinary science and humanities training. The latter three years are clinical clerkships focused on clinical medicine. Medical students enter their clinical clerkship with bioethics, resilience, communication, and professionalism training, which helps them develop their moral compasses [25,26]. 

### 2.2. Study Design

This study employs an explanatory mixed-method sequential design [27]. The first quantitative phase began with a cross-sectional survey, assessing the prevalence of witnessing or participating in professionalism dilemmas they think unethical during clerkship and examining the moral distress resulting from these experiences. In the subsequent qualitative phase, the semi-structured interviews were adopted as follow-up inquiries to help explain survey findings and explore other professionalism challenges and related emotions and reactions during the clerkship. Results from both phases were integrated and triangulated during the discussion, which enriches the study by confirming and refuting findings on how the two phases shared similarities and differences.

### 2.3. Participants

#### 2.3.1. Survey Participants

All medical students enrolled in the institution, who have exposure to clinical rotation, were invited to participate in the study. Eligible respondents were asked via their institutional email with a link to the survey questionnaire. Participation was voluntary. A study introduction stated on the first page of the survey that participants were informed that the participation was voluntary, and their responses would remain anonymous. Electronic consent was obtained from participants before accessing the survey. After the initial invitation, two reminders were sent every other three weeks.

#### 2.3.2. Interview Participants

Out of 153 survey respondents, 45 (29.4%) consented to participate in the interview and left their contact information. An interview invitation was sent to all who agreed to a follow-up interview. At last, nine responded to the invitation: four students from Year 4, three from Year 5, and two from Year 6. 

During the consent taking, participants were reminded that study participation is voluntary, and they can withdraw from the study at any time. Interviews were conducted without audio or video recordings. Researchers (C.C. and W.Y.K.K.) took the minutes of the discussions and completed the field notes a week after each interview. Each interview lasted between 30 and 50 min. Upon completion, participants received a book voucher for HK$200 (US$25) as a token of appreciation.

### 2.4. Study Instruments

#### 2.4.1. Survey Measures

The quantitative study objective is to (1) assess the prevalence of witnessing or participating in professionalism dilemmas that they think are unethical during clerkship and (2) examine the level of moral distress resulting from these experiences.

A survey was developed and modified based on Wiggleton and colleagues’ study [1], examining experiences of professional dilemmas and distress conducted in Anglo-European settings. Drawing reference from common dilemmas observed by medical students, several items, including sexual harassment, inadequate care, student abuse, consent, confidentiality, and intimate examination, were also incorporated into the survey [6,11,28]. In the development of the final 30 professionalism dilemmas, investigators (C.C., W.Y.K.K., and O.M.Y.N.) discussed the themes and wording of survey items that are acceptable and appropriate in the local context. A senior student with two years of clerkship experience and a physician leading the professionalism teaching modules (W.T.W.) were involved in reviewing the items and assessing the face validity and content validity, respectively.

Table 1 describes the 30 dilemmas grouped under four themes, including involvement in care perceived to be substandard (5 statements), professionalism lapses (8 statements), responsibility exceeding students’ capabilities (10 statements), and system constraints (7 statements). Based on the 30 dilemmas, respondents were asked to report whether they had observed or experienced any of these dilemmas in the past 12 months. They were invited to rate the frequency of its occurrence using a five-point Likert scale from never, infrequently (1–2 times per year), occasionally (1 time per 2 months), frequently (1 time per month), and very frequently (2+ times per month). Using the same scenario, respondents were asked to rate the level of distress caused by these dilemmas. If the respondents had not encountered the dilemmas, they were invited to answer the questions hypothetically and rate their perceived distress. A four-point scale was adopted from no distress, mild distress, moderate distress, and severe distress.

#### 2.4.2. Interview Guide

A semi-structured interview guide was designed to explore professionalism dilemmas encountered by the students and the subsequent emotions and reactions during clinical clerkship (See Table 2). Interviewees were invited to share (1) dilemmas or wrongdoings at the workplace and (2) their responses to the problematic dilemmas.

### 2.5. Data Analysis

#### 2.5.1. Quantitative Analysis

IBM SPSS Statistics for Windows, version 27 (IBM Corp., Armonk, NY, USA) was used to analyze the data, including the frequency of witnessing or participating in 30 dilemmas and perceived distress levels. Cronbach alpha was calculated to measure the internal consistency of individual themes. Values of 0.7 or higher indicate an acceptable internal consistency [29]. 

#### 2.5.2. Qualitative Analysis

Thematic analysis [30] was adopted to understand dilemmas in which medical students were disturbed and the students’ coping reactions. Reflective journals and interview notes were triangulated and analyzed by two independent researchers (C.C. and W.Y.K.K.) to get a general impression of the data. The representative texts were then labeled inductively with open axial coding focusing on the latent interpretation. A constant comparative method of coding was used to create the themes. New thematic codes that did not fall into predetermined categories were developed and refined. The codes were compared and discussed among research members until a consensus was reached. Member checking with experts (O.M.Y.N. and W.T.W.) was conducted to increase credibility and allow the transferability of findings to real-life settings.

## 3. Results

### 3.1. Survey Findings

#### 3.1.1. Demographic Characteristics of Survey Respondents

Table 3 describes the demographic characteristics of survey respondents. A total of 153 students completed and returned the survey, with a response rate of 21.7% (153/706). Most were final-year students (82.4%) and female (52.9%). The average age is 23.37, ranging from 20 to 31 years old. 

#### 3.1.2. Frequency of Professionalism Dilemmas Encountered by Medical Students

**Figure 1:**
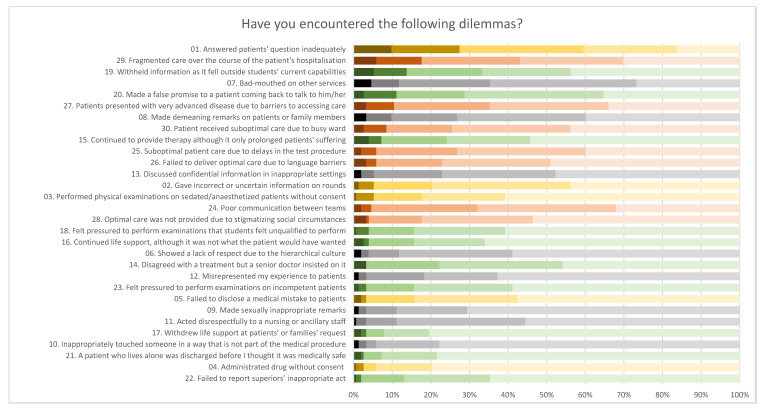
Frequency of professionalism dilemmas encountered by medical students. (1) Bar color indicates the theme of the measures: yellow = involvement in care perceived to be substandard; gray = professionalism lapses; green = responsibility exceeding students’ capabilities; orange = system constraints. (2) Dark and light color palette (From left to right): very frequently, frequently, occasionally, infrequently, and never.

The four scales show high reliability: involvement in care perceived to be substandard (α = 0.848), professionalism lapses (α = 0.863), responsibility exceeding students’ capabilities (α = 0.901), and system constraints (α = 0.922). Figure 1 shows the frequency of professionalism dilemmas medical students have encountered. The top five most frequently occurred dilemmas were the team answering a patient’s questions inadequately (27.5%), observing a patient who received fragmented care (17.6%), withholding information from a patient who requested it because the students felt it was not their responsibility or place to provide it (13.7%), bad-mouthing other departments (11.8%), and making a false promise to a patient that the students will come back to talk to him/her (11.1%).

The most infrequently experienced or that never occurred scenarios were observing dilemmas where a ward mate touched a patient, family member, other staff, or a student inappropriately that is not a part of any necessary medical procedure (94.1%), and the drug was administrated without patient’s consent (94.1%). Followed next was observing a patient discharged before a student thought it was medically safe (92.8%), withdrawing life support at the patient’s or family’s request (92.1%), acting disrespectfully to the nursing or ancillary staff (88.9%), and making sexually inappropriate remarks (88.9%). 

#### 3.1.3. Perceived Level of Moral Distress in the Professionalism Dilemmas 

**Figure 2:**
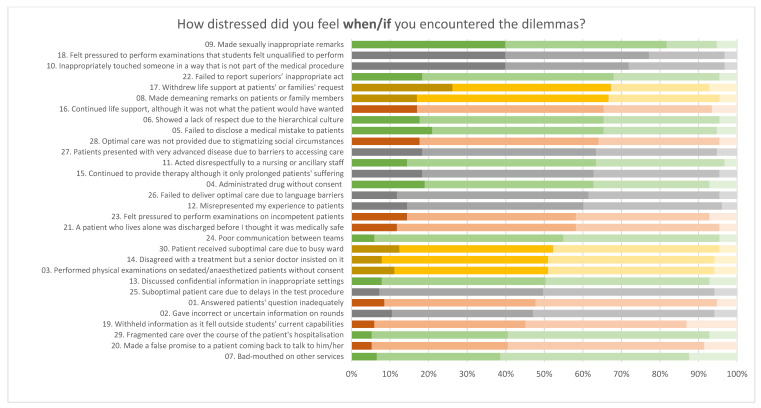
Perceived level of moral distress in the professionalism dilemmas. (1) Bar color indicates the theme of the measures: yellow = involvement in care perceived to be substandard; gray = professionalism lapses; green = responsibility exceeding students’ capabilities; orange = system constraints. (2) Dark and light color palette (from left to right): severe distress, moderate distress, mild distress, and no distress.

Figure 2 shows the level of moral distress in the professionalism dilemmas. The majority of respondents felt moderately to severely distressed when they observed a ward mate make sexually inappropriate remarks (81.7%), were pressured by a senior doctor to perform a procedure they did not feel qualified to do (77.1%), observed a wardmate touching a patient, family member, other staff, or a student inappropriately that was not as a part of any necessary medical procedure (71.9%) and failed to report superiors’ inappropriate act (68.0%).

### 3.2. Interview Findings 

Nine interviews were conducted after the survey results were collected: four students from Year 4, three from Year 5, and two from Year 6. The following sections present the two major themes: dilemmas or wrongdoings at the workplace and coping strategies in response to difficult dilemmas.

#### 3.2.1. Dilemmas or Wrongdoings at the Workplace

Four general recurring sub-themes emerged and were categorized by the stakeholder’s interactions, namely (i) doctor-to-patient, (ii) doctor-to-student, (iii) student-to-patient, and (iv) student-to-student.

(i) Doctor-to-Patient Interaction

From the perspective of medical students who first enter their clinical years, observations of colleagues at the workplace occurred at a physical and psychological distance from oneself. This distance could exist because the students know they are not qualified healthcare professionals yet and hope to fit into the role of a doctor-to-be in a clinical setting. There is a clear hierarchical divide between their ward group and the practicing healthcare professionals.

*Verbal Abuses*

All interviewees witnessed a doctor use disrespectful words or language towards patients. There were three general forms of verbal abuse: rude name-calling, lack of empathy towards a patient, and ignoring a patient.

*“I have seen patients being referred to with “fucked up names like ‘vege vege (choy choy)’ if they are vegetative state. That’s a lack of respect. Or they were calling a patient ‘it,’ like ‘it is a case of cerebral palsy.”*

(Interview 9, M23, O)

When asked whether or not calling a patient “morbidly obese” is disrespectful, there was a consensus that it was acceptable as long as the comment was made to improve the patient’s health.

*“Talking about the truth is okay. It is okay if they have a reason behind why they are telling them they are fat. The patient might not understand the other words [medical jargon].”*

(Interview 4, M24, M)

*“It is important to let them know. Do not avoid it. It is about how you deliver the message: like ‘clinically overweight and associated health risks’—it’s how they take it: medical advice or judgment? Plus, “obese” in Cantonese is a medical term. So, it is appropriate and encouraged if it is for their health.”*

(Interview 3, M24, M)

A few had mixed opinions about using the term “obese” toward patients.

*“I am very numb to it, and it is not on my radar anymore. Both are unnecessary, whether calling the patient obese out of concern for health or talking to someone else nearby. Nine out of 10 doctors will say this to a patient, and I’m not sure I’m offended.”*

(Interview 9, M23, O)

*Active Listening and Empathy are Absent*

A lack of empathy and attention to the patient’s feelings disturbs students. This occurred in various settings, including outpatient clinics and during ward rounds. There is a consensus that both the failure to empathize with patients in outpatient clinics and ignoring patients’ requests when passing them by in the wards are morally wrong. Still, the latter was expressed repeatedly by multiple interviewees as a very commonly seen behavior.

*“I was in an abortion clinic, and the doctor did not give any counseling. It was abrupt, saying that ‘the baby’s gone, and this is what you have to do XYZ,’ you could see the patient was emotional, but the doctor did not address the emotions. The management of a sensitive issue requires more care in communication. It’s important to address the emotional part.”*

(Interview 8, M23, F)

*“I was in a cardiology outpatient clinic. The doctor was transiently empathetic and more pragmatic about the problem than consoling. The patient was on the verge of tears, but the doctor just talked about social support and practical issues, saying there was nothing he could do to help the patient.”*

(Interview 4, M24, M)

*“If the patient calls the doctor and there is no response, it is normal. The requests cannot be fulfilled, or it is not your duty. They should get a response, but many times there is no response and pretend they [doctors] do not hear them [the patients].”*

(Interview 7, M23, F)

*Unconsented Physical Examination of Patients*

Interviewees observed some physical interactions between doctors and patients that compromise physical safety or directly affect patients through unwanted touch or physical examination. For example, doctors were performing or instructing students to perform physical examinations on an unwilling patient or one who is unable to consent occurred the most in the interviews.

There were also instances of patients not being asked if they consented to a particular examination, i.e., physical examinations proceeding without informing them.

*“A doctor did a transvaginal exam suspecting a mass and immediately afterward did a transrectal exam without informing the patient. The patient was like, ‘Oh what? That’s not my vagina’. In practice, the only ‘verbal consent’ between doctors and patients is usually ‘I’m going in now,’ and the patient will be silent or just be like ‘okay.’”*

(Interview 9, M23, O)

According to one student, a patient who directly refused an examination was challenged by the doctor in question, who wished for his students to conduct the physical examination regardless of the non-consent.

*“The doctor pressured a male patient to allow medical students to let them listen to the lung, and when the patient said no, the doctor said, ‘are you not feeling well?’ I felt a little uneasy but still quickly listened to the lung.”*

(Interview 5, M22, M)

Students felt distressed as the intrusive examination was performed without proper consent, which was heavily emphasized during pre-clinical ethics teaching. Nearly all interviewed students mentioned the inappropriateness of performing physical examinations on patients who refused or were incapacitated to consent to the procedure. Examples included practicing physical examinations on patients with dementia, under anesthesia, or patients who had explicitly refused to be examined. One student recounted a case of a per rectal examination being performed on a patient who was under general anesthesia.

*“Even a consenting patient usually does not consent to the rectal exam, so it’s very inappropriate for the medical student to do it on a patient who is also ‘out of it.’ To some extent, performing a physical examination benefits the student. In my opinion, it was inappropriate for an unresponsive patient to undergo it repeatedly without their consent. I feel that the medical students’ learning should benefit the patient.”*

(Interview 7, M23, F)

Another interviewee believes that different consent standards exist in other specialties due to the capacity of understanding (pediatrics vs. adult patients) or degree of intimacy of the body part being examined, such as the genitals.

*“In pediatrics, many children are bed-bound and cannot speak. You still have to do it as this is the disease [that they cannot speak/consent]. They have a higher standard in urology, doing DRE, or in OG, relating to your private parts [of consent]. OG has a high standard of consent, and they explain very well and tell you [about the procedure] many times. They should all have the same standard for everything in an ideal world.”*

(Interview 7, M23, F)

*Physical Restraints on Patients*

Another topic within the subject of consent is the issue of restraints placed on patients. The interviewees in Year 6 were especially vocal about situations in which some patients get restrained if they have pulled out drips or a catheter or been agitated or aggressive.

*“It’s not good because the patient cannot be mobile, especially for the elderly. They can’t drink water, which leads to dehydration; this affects the patient. Sometimes the doctor agrees [to restrain the patient], even if they don’t know the reason.”*

(Interview 5, M22, M)

*Bias in Clinical Settings*

Discriminations toward patients from healthcare professionals, based on reasons irrelevant to their clinical condition, could be considered verbal abuse, depending on the situation. Specific laboratory tests were ordered for certain patients based solely on their race but not medical reasons. It can thus be physically intrusive or unnecessary to the patients and morally distressing to students.

*“I asked why we needed to do the test for parasites just out of curiosity, but the doctor said ‘because the patient was that ‘type’ of people. I found it disappointing because the doctor was otherwise really nice, and [they] enjoyed the out-patients clinics until that point.”*

(Interview 9, M23, O)

In addition, ignoring patient requests out of discrimination translates to neglecting their physical needs. Still, it can imply that a morally distressing verbal situation occurred because a vocal request was not addressed.

*“The doctor or healthcare staff get impatient when [patients] can’t explain [their needs] in [Chinese or English]. Although it’s better to write or draw something out, it doesn’t happen all the time. Translation services aren’t always available either.”*

(Interview 2, M24, F)

(ii) Doctor-to-Student Interaction

*Inappropriate Comments Made by Teaching Doctors*

Students acknowledged the existence of a relationship between teaching doctors and patients and between doctors and students. Depending on the delivery and tone, doctors’ responses to students getting wrong answers can be troubling. Implications of harsh comments can discourage students, who do not learn anything new from the interaction but feel poorly about themselves. Doctors have been reported to imply that the student will or has failed this year already and even been told to commit suicide, apparently as a joke.

*“[The doctor said] ‘oh, you have already given up? It’s a long time until next year!’”*

(Interview 6, M22, M)

*“Objectively, I have seen things that would be considered disrespectful, but students who know him would think he’s joking. When one student does one thing wrong in physical examination, he would say it doesn’t matter; go jump out the window. The ward group […] didn’t laugh along.”*

(Interview 4, M24, M)

Students found comments unrelated to education inappropriate. If the statement was made regarding academics, it was acceptable and unacceptable if not related to academics

*“[The difference is] whether they’re trying to teach you something or not. Commenting on anything other than your ability is disrespectful.”*

(Interview 8, M23, F)

One interviewee recalled a troubling dilemma in which a surgeon made sexual remarks to a female medical student in the operating theatre.

*“While doing the mastectomy, the doctor pointed to the [female] ward mate, who said she looks like a transgender male. […] He teased that the ward mate must be familiar with mastectomy and have done it in Thailand.”*

(Interview 1, M24, M)

The student was further disturbed by the comment made during a breast surgery on an unconscious, anesthetized patient, which shows that the context plays a role in the level of distress experienced. Perhaps the student wondered if the patient could hear the comment and feared that she would be offended that her surgery was being done to remove a cancerous lesion, as compared to a breast augmentation surgery. Other more subtle comments made toward students were also reported.

*“I think there has been sexual harassment like calling me pretty by a professional.”*

(Interview 8, M23, F)

(iii) Student-to-Patient Interaction

*Inappropriate Verbal Remarks*

The recurring examples were witnessing classmates label patients by their diseases and make sexually inappropriate remarks in clinical settings. One student recounted seeing a classmate referring to a pediatric patient with Down’s syndrome by his disease and not by his name.

*“In pediatrics, upon some children with Down’s Syndrome, I heard a ward mate use a teasing attitude to call the patient, ‘this is a Down’s.’ Using the diagnosis to label the patient is inappropriate. I felt that the ward mate did not have bad intentions and was just making a joke, but it’s a form of disrespect.”*

(Interview 6, M22, M)

The student did not report the incident in this instance due to the fear of fracturing the relationship between ward mates, who must remain on the same team throughout the academic year.

*“If I were close with the ward mate, I would tell him not to call a patient by their diagnosis even as a joke, but we were not close, so I did not say anything. The reason is that my ward mate and I will cooperate for a year and would not want to compromise the relationship.”*

(Interview 6, M22, M)

Another interviewee expressed frustration at the behavior of a verbally offensive ward mate. During a gynecological rotation, the doctor performed a transvaginal ultrasound on an elderly lady with dementia. The doctor apologized to the student that it looked like he was raping her, to which the male student responded with, “it’s okay, and we’ll just watch you rape her”. The interviewee commented that:

*“This guy [the male student] is misogynistic; he makes offensive comments. Then the whole room went silent. He apologized in front of everyone and then to the doctor afterward, but both comments were bad.”*

(Interview 9, M23, O)

Students also experienced distress when performing physical procedures that they felt incompetent on patients. A final-year student recounted drawing blood on patients for the first time but had to do so without the doctor’s supervision as the doctor was busy addressing other issues.

*“Taking blood can only be done when the doctor is present, but the technician said that even if the doctor is not here, you can do it, so they technically didn’t follow the rules despite being instructed to do so.”*

(Interview 5, M22 M)

*Privacy Breaches*

One student found it distressing to see a ward mate disregard patient privacy.

*“I’ve seen him take pictures of the operation theatre list without covering the personal details, and defended himself by saying his iPad is encrypted.”*

(Interview 3, M24, M)

In this case, the student reported the ward mate for repeated infractions regarding patient privacy and clerking female patients without a chaperone.

(iv) Student-to-Student Interaction

*Sexual Harassment between Ward Mates*

Although only one interview documented significant student-student tensions, it is still of much value to analyze due to the seriousness of the incident. This interviewee recounted events regarding a ward mate that created conflict and tension within the group.

*“I have a ward mate who does not respect personal boundaries. He would stand very close to ward mates and touch them or stand very close to them. He touched a female war mate’s butt twice, touched their hands, and stood close while looking at an X-ray. Since September, and I feel it’s very problematic.”*

(Interview 3, M24, M)

In this ward group, inappropriate touching or disrespecting physical boundaries occurred over several months. There was a lengthy discussion among the members of the ward group concerning why the perpetrator acted this way, and he even came up with a “conspiracy theory” after the initial complaint.

*“Later in the fall, the ward mate in question reportedly stood too close or touched both boys and girls, but only after a big complaint from the girl, so now there’s a conspiracy of him doing that so that they can’t say he only targets girls.”*

The interviewee did not formally report the dilemma and discussed the matter informally with friends. He did not take action because the victims did not want to report it, and he felt he would disrespect their wishes.

*“Although [the girls] were disturbed by [the ward mate’s] actions, they did not wish to report it. I think it’s because they thought [the ward mate] would have huge repercussions.”*

*“It did not feel right to intervene and that it would be strange for me to do this alone … I would feel okay about it if I did it for the girls [if they asked me to].”*

As the interview proceeded, the interviewee revealed his genuine opinion that the action of inappropriate touching was not truly sexual harassment because of his perceived lack of legal ground in Hong Kong. However, he also expressed a contradictory view that the incident was still worth reporting. Thus, perhaps his doubt of the incident’s severity contributed to the lack of action.

*“I would take the victim’s point of view, but it was still borderline sexual harassment since the case would be lost in court. I felt burned out and saw a counselor about these situations. I would be very burned out if [the situations] repeat a lot. The support I’ve is okay for now [from the] Wellness Centre for emotional support.”*

#### 3.2.2. Coping Strategies in Response to the Difficult Dilemmas

##### Students’ Inaction in Dilemmas

None of the interviewees made defiant actions or reported the incidents. Reasons this occurred include fear of breaking down the doctor-student relationship. Students also worried about the potential impact on their career prospects in the profession from their decisive actions against the encountered unprofessional scenarios.

*[A student whose female ward mate received a comment about being transgender with breast augmentation surgery] “I would not report this personal attack because I’m a student and he’s a trained doctor; if I had a chance to work with him in the future, reporting the doctor might impact my career, and give other people the impression that ‘this guy is a whistle-blower,’ it leaves a bad reputation for him.”*

(Interview 1, M24, M)

*[Situation in which the patient was unaware that the doctor was going to perform a per rectal exam after the vaginal exam] “The doctor was yelling at the students afterward, so there was the feeling that she wasn’t going to be kind to either patient or student, and the students didn’t speak up.”*

(Interview 9, M23, O)

Students perceived some troubling situations as ambiguous or not severe enough to take action and even doubted the appropriateness of their distress feeling. As a result, they were more likely to talk to the victim in question rather than to confront the perpetrator in these situations.

*“I don’t know when it’s disrespectful and what’s being too sensitive, but when I tell my friends, they are surprised by it.”*

(Interview 9, M23, O)

*“A doctor was directly yelling at [my ward group], calling us stupid, but I was not sure if the doctor was being funny, judging by his tone, or felt superior and entitled to do so.”*

(Interview 5, M22, M)

*“I would ask […] if she’s okay in private, but I would not confront the doctor, if the ward mates are not feeling okay then talk it out.”*

(Interview 1, M24, M)

*[Students who were told to “jump out the window” by a doctor] “[My ward mates] didn’t get upset, but we talked about it and thought it was inappropriate.”*

(Interview 4, M24, M)

Some students felt that harsh comments from doctors towards students might be part of the natural process of medical education, and these comments are usually related to education. However, while some students find these comments with harsh tones, even “yelling,” acceptable and not disrespectful, others find them unnecessary since it does not benefit them in terms of learning.

*“You see so many of these [situations], so maybe it’s part of [medical] education.”*

(Interview 9, M23, O)

*“This is how it is … but really? Is it even helpful? It did not help me remember [the material I was scolded for].”*

(Interview 8, M23, F)

##### Helplessness in the Hierarchical Health Care System

Students felt powerless about the systemic issues behind the problems. They mentioned “authority,” “hierarchy,” and “rank” when explaining why they had not refused to perform the physical examination if they felt uncomfortable. However, they expressed their enthusiasm to change the culture once they got the status.

*“It’s part of the culture. If you see someone do this, you will do it too; it’s quite convenient, but if a nurse were to ask me to sign to restrain a patient when I become a doctor, I would say no.”*

(Interview 5, M22, M)

*“The hierarchy of the doctor being the one with the most authority is a big component [of why I went along with the physical exam]. It would have been awkward if I had said something.”*

(Interview 5, M22, M)

*“I’m nobody; I cannot change this situation unless everyone has an awakening of conscience that restraining without proper reason is bad.”*

(Interview 6, M22, M)

##### Students’ Reflection on Uneasy Situations

A theme that emerged was students’ reflections on these morally distressing dilemmas. Two opposite views were noted. Some did not feel burnout or disillusionment from moral distress but instead saw them as learning experiences.

*“I don’t think I’ll burn out, but I will be more tactful when dealing with patients, try to be less blunt”*

(Interview 1, M24, M)

“I will be much more reluctant to put patients in restraints in the future” (Interview 5, M22, M). Other students reported negative consequences from experiencing moral distress, including a decline in their code of ethics, feeling desensitized, and compassion fatigue. In response to how she felt about calling patients disrespectful names, a student responded that:

*“It’s common to call them [patients] derogatory terms like “obese” to the point that I’m desensitized. I’m to some degree desensitized, but at the beginning, I was shocked. I would say it in the future as I’m desensitized now. As a friend, it’s not acceptable, but it’s accepted in the ward environment”*

(Interview 7, M23, F)

##### Practical Application of Knowledge Learned from Professionalism and Bioethics Classes

Regarding their opinion on the professional and bioethics teaching before and during their clerkship, five out of nine students felt it helped establish a foundation for the future application of the principles. In contrast, the other four students thought that practical advice based on real-life experience was more helpful than theoretical teaching.

*“To be honest, not much can be applied from the bioethics course. Usually what the doctor says from experience and practical advice is more helpful.”*

(Interview 5, M22, M)

*“As a medical student, the four principles are less used, maybe when there is decision-making, e.g., when to admit what patient in the ICU; if there are not enough beds, then when to discharge patients, they would be more relevant.”*

(Interview 5 M22, M)

Several students felt more support was needed to help them cope with their distress when encountering dilemmas, including practical interacting teaching in professionalism and bioethics classes, a clear and accessible reporting and feedback system, and good role modeling by compassionate physicians to create a culture of welcoming ethical discussions of moral dilemmas.

*“[What is missing is] good role models, who will not call a patient ‘it’ and show you ‘this is how to make a patient comfortable.’ When they explicitly state these things and show that they prioritize warmth, people become more compassionate. A long-winded doctor will go through consent.”*

(Interview 8, M23, F)

## 4. Discussion

The present study’s findings provided evidence that medical students frequently encountered dilemmas they felt uncomfortable with and experienced distress to a varying degree as a result. Our study revealed the top two encountered dilemmas were observing a physician failing to respond to patients adequately and patients receiving fragmented care provided by the healthcare team. In congruent with the qualitative analysis of the interviews, doctor–patient interactions, namely observed doctors’ verbal abuses towards patients, physical examinations without proper consent, order of unnecessary restraints on uncooperative patients, bias clinical decisions based on non-clinical reasons, were the most prominent recurring themes if categorized the emerged themes by stakeholders’ interactions.

The high frequency of reporting problematic interactions between doctors and patients may be due to the severe shortage of doctors. The doctor–population ratio in Hong Kong, 2.0 doctors per 1000 population, is far below Singapore’s 2.5, the United Kingdom’s 3.0, and Australia’s 3.8 [31]. In situations where overwhelmed physicians are forced to prioritize tasks, appearing to ignore patients may be more common or perceived acceptable due to systemic constraints.

### 4.1. The Incongruity between Medical Education and Patient Care

Failure to obtain informed consent is a rare instance reported in the survey but a recurring theme in the interview. This is not exclusive to our study and is also frequently the case in the relevant literature [6,32]. The primary examples were doctors performing or instructing students to perform physical examinations on non-consenting patients. As a result of the professional lapses, students questioned the training-practice discrepancies. In this instance, they pondered on the importance of consent, whether it truly varies in different clinical contexts, and the correlation between ethical theories of informed consent and societal values influenced by the Chinese cultural norms in Hong Kong.

Regarding the influence of culture on a society’s view on consent, perhaps patients in Hong Kong are used to a patriarchal healthcare system. They believe doctors know best, so hesitant consent is, if not acceptable, at least not a “reportable” issue. However, there were no instances where patients were observed to report a non-consenting physical examination, so it is unclear whether a report would be accepted. Another possible reason these instances are not reported is that no “real” or bodily harm was done, and they believe there will be no proof after it occurs. An additional study investigating how the average citizen reacts to observing a physical examination conducted without consent would shed light on the public’s opinion on the adequacy of informed consent. It would not be surprising that physical examinations on parts of the body considered more intimate in this society would correlate with a perceived higher standard of consent required.

### 4.2. Students’ Inaction in Professionalism Dilemmas

Role modeling with exemplary behavior in clinical settings was a dominant factor influencing how students construct a professional identity [33]. The quality of role-modeling varies and fails because of interactions with teaching faculty that the students consider hostile or unwelcoming towards them. The professionalism dilemmas not only desensitize students from the prior idealized notion acquired from pre-clinical teaching but also force their acceptance of authentic medical culture. In our study, students commented that these experiences serve as negative examples reminding them not to repeat the action themselves in the future when they are a doctor.

Reactions to the professionalism dilemmas are minimally observed, despite the distresses. The medical hierarchy and power dynamics between doctors and students contributed to the students’ inaction. The fear of defying the higher status of the doctor, breaking down the doctor-student relationship, and impacting their future career are a few student-specific concerns that led to their inaction [34]. Studies showed that moral distress contributed to lower quality of patient care, cynical attitudes [35,36], diminished empathy [37], and accumulated over time and led to new morally stressful dilemmas [34]. A responsive reporting system, authority figure, and follow-up mechanism were facilitators in acting on professionalism dilemmas [38].

### 4.3. Sexually Inappropriate Remarks in Medical Education

Sexual harassment and mistreatment are pervasive problems in medicine and remain prevalent [39]. The frequency of sexually inappropriate remarks is low in the survey results. However, it was the second-highest cause of severe distress if or when a student encountered the situation. Similar to previous studies [40,41], interviews reported that verbal and non-verbal experiences were offensive, and sexually suggestive comments about one’s physical appearance were distressing. Sexual assault was not reported in our study, though one interviewee has recounted an accusation of sexually inappropriate bodily contact, and it is not uncommon in the literature [42]. Gender (female), sexual orientation (non-heterosexual orientation), and race/ethnicity (non-white population) were more vulnerable to sexual harassment than their counterparts [43]. Some victims reported academic disengagement [43,44], and perceived diminished choice of specialty after graduation [45]. Promoting awareness and training to prevent sexual harassment upstream is the key to avoiding a hostile learning environment. Bi-directional concrete interventions could be taken to diminish sexual harassment effectively [46]. From the bottom-up level, education sessions shall be implemented for students and faculty members to help maintain sexual and professional boundaries. From the top-down level, the Faculty shall strengthen the mechanism for handling sexual harassment cases.

## 5. Study Strengths and Limitations

Our study reports that undergraduate medical students in Hong Kong are exposed to professionalism dilemmas with moral distress, possibly in relation to the system constraints, organizational culture, peer influence, and inadequate demonstration of ethical knowledge applications by clinical teachers. The findings highlight the need to implement strategies responding to unprofessional and unethical behaviors by teachers and students in the medical education environment.

Data interpretation should be made carefully due to some methodological limitations. Firstly, this study was conducted during COVID-19, where clerkship training was suspended in lieu of stringent social distancing measures. The findings may be underreported and may not be generalizable to the conventional learning environment. Secondly, the study involved a scoping survey based on 30-item dilemmas using a retrospective design that relied on recalled estimates. Finally, the respondents were sampled from one tertiary medical school and involved a small sample size for the survey (*n* = 153) and interview (*n* = 9) only. The study is limited by a response bias. The interviewees who agreed to participate in the survey and interview may appeal to the topic more than those who responded and answered in a socially desirable manner. However, we ensured that no repercussions would be caused to the medical students at the beginning of the survey or interviews to seek their honest feedback and sharing. Findings were analyzed with subjectivity shall be generalized carefully in light of a small number of interviewees. We plan to conduct a longitudinal study to assess the frequency and level of moral distress in the face of professional dilemmas as student advances to senior years.

## 6. Conclusions

This study revealed that witnessing suboptimal doctor–patient interactions when doctors inadequately communicate with the patients and touch or instruct students to examine the patients without proper consent were the most common professionalism dilemmas encountered by medical students in their clinical clerkship. Despite the degree of reported moral distress, medical students were reluctant to put forward a strong reaction toward the unprofessional behaviors they encountered. Findings ascertained that students experienced problematic situations within the realm of bioethics and professionalism during the clinical clerkship. Two educational implications could be drawn: (1) the importance of continuing bioethics and professionalism teaching incorporating a reporting mechanism for unprofessional behaviors, and (2) the essence of role modeling by the teaching faculties to improve the learning environment for the medical students. Further studies should be conducted to understand better how students can better cope with these moral dilemmas and how to implement a truly anonymous reporting system for students.

## Figures and Tables

**Table 1:** Themes and descriptions of the 30 dilemmas.

	Themes	Descriptions
	Involvement in Care Perceived to be Substandard	
01	Answered patients’ questions inadequately	The attending physician or resident answered patients’ questions inadequately, e.g., rushed through the consultation or simply ignored them
02	Gave incorrect or uncertain information on rounds	A member of my ward group/consulting team gave incorrect or uncertain information on rounds
03	Performed physical examinations on sedated/anaesthetized patients without consent	The consulting physician performed examinations on sedated or anaesthetized patients without their consent
04	Administered drug without consent	The consulting physician administered drugs without obtaining the patient’s consent
05	Failed to disclose a medical mistake to patients	An error was made in the care of a patient that was not fully or truthfully disclosed
	Professionalism Lapses	
06	Showed a lack of respect due to the hierarchical culture	A member of my ward group/consulting team was disrespectful to someone below himself or herself on the team ranking, e.g., student or other staff. (Disrespect defined as: receiving or witnessing covert, status-related abuse, verbal abuse, physical abuse, and ⁄ or harassment or discrimination, e.g., sexual, racial, religious, age, gender)
07	Bad-mouthed other services	Members of my ward group/consulting team “bad-mouthed” other services, e.g., other specialties, departments
08	Made demeaning remarks towards patients or family members	A member of my ward group/consulting team made disparaging or demeaning remarks about one of our patients or their family members. (Examples of “disparaging/demeaning remarks”: any kind of unnecessary judgment towards the patient, e.g., commenting on weight (“You’re too skinny/fat!”), commenting on behavior/lifestyle in a disparaging way (“Are you trying to kill yourself by [behavior]?” “Who raised you?”), commenting on the appearance in an unnecessary or unprofessional manner (“You look sickly.”)
09	Made sexually inappropriate remarks	A member of my ward group/consulting team made sexually inappropriate remarks about a patient, family member, other staff, or a student, e.g., flirting, giving inappropriate comments about physical appearance
10	Inappropriately touched someone in a way that is not part of the medical procedure	A member of my ward group/consulting team touched a patient, family member, other staff, or a student inappropriately, i.e., not as a part of any necessary medical procedure
11	Acted disrespectfully to a nursing or ancillary staff	A member of my ward group/consulting team was disrespectful to the nursing and/or ancillary staff
12	Misrepresented my experience to patients	A senior doctor misrepresented the degree of my experience in performing a procedure to the patient
13	Discussed confidential information in inappropriate settings	Members of my ward group/consulting team discussed confidential information about the patient in an inappropriate setting, e.g., public spaces, with friends or family, on social media, or messaging apps
	Responsibility Exceeding Students’ Capabilities	
14	Disagreed with a treatment but a senior doctor insisted on it	My ward group/team went along with a treatment that we did not believe was indicated, because a senior doctor insisted on it
15	Continued to provide therapy although it only prolonged patients’ suffering	I witnessed that staff/my consulting team continued to provide therapy, even though I thought it only prolonged the patient’s suffering
16	Continued life support, although it was not what the patient would have wanted	I witnessed that staff/my consulting team continued life support, even though I thought it was not what the patient would have wanted
17	Withdrew life support at patients’ or families’ request	I witnessed that staff/my consulting team withdrew life support at the patient’s or family’s request, even though I thought the patient could have survived with continued treatment
18	Felt pressured to perform examinations that students felt unqualified to perform	I performed a procedure that I did not feel qualified to do because I was afraid of being perceived as incompetent or I felt pressured by a senior doctor
19	Withheld information as it fell outside students’ current capabilities	I withheld information from a patient who requested it because I felt it was not my responsibility or place to provide it
20	Made a false promise to a patient coming back to talk to him/her	I promised one of my patients that someone would come back to speak to him or her, even though I was not sure it would actually happen
21	A patient who lives alone was discharged before I thought it was medically safe	A patient was discharged before I thought it was medically safe because there was no one at home to care for the patient
22	Failed to report superiors’ inappropriate act	One of my superiors behaved inappropriately, but I did not report it because I was afraid of negative consequences, e.g., it would affect my evaluation, or because I was not confident that I was right
23	Felt pressured to perform examinations on incompetent patients	I performed examinations on a patient who was incompetent (e.g., a minor, sedated, or had a mental disorder) because I felt pressured by a senior doctor
	System Constraints	
24	Poor communication between teams	Poor communication between multiple consulting teams that negatively affected his or her care
25	Suboptimal care due to delays in the test procedure	Delays occurred in the performance of tests or procedures, or the return of laboratory data or radiology reports because of scheduling problems or lost requests. Such delays resulted in suboptimal patient care
26	Failed to deliver optimal care due to language barriers	Optimal care was not provided to one of my patients (e.g., ethnic minorities) because of language barriers
27	Patients presented with very advanced disease due to barriers to accessing care	A patient presented with a very advanced disease because he or she faced barriers to accessing care
28	Optimal care was not provided due to stigmatizing social circumstances	Optimal care was not provided to a patient as a result of stigmatizing social circumstances or conditions (e.g., age, alcoholism, drug abuse, homelessness, religion, or obesity)
29	Fragmented care over the course of the patient’s hospitalization	Over the course of a patient’s hospitalization or long-term treatment, he or she was cared for by multiple doctors and services, which led to fragmented, discontinuous care. E.g., Roles were not explained adequately, or the patient had to explain their condition repeatedly
30	Patient received suboptimal care due to busy ward	Suboptimal care was provided to a patient because my ward group/consulting team was too tired and overworked

**Table 2:** Sample Interview Questions.

Questions
Have you ever encountered professionalism dilemmas during the clinical clerkship in the past 12 months? Please share with us some examples. How did you react or feel, and what did you do in response to the dilemma(s)? Have you discussed the incident(s) with your peers or teachers? How would this/these incident(s) affect your future clinical practice? How would bioethics or professionalism workshops help you cope with the dilemma(s) or negative emotions?

**Table 3:** Demographic characteristics of survey respondents.

Demographics	*n* (%)
Class Year	
Year 4	19 (12.4%)
Year 5	8 (5.23%)
Year 6	126 (82.4%)
Gender	
Female	81 (52.9%)
Male	68 (44.4%)
Non-binary	2 (1.3%)
I prefer not to say	2 (1.3%)
Age	
Mean	23.5
Range	20–31

## Data Availability

The data supporting this study’s findings are available from the corresponding author upon reasonable request.
